# Advancing translational research in neuroscience through multi-task learning

**DOI:** 10.3389/fpsyt.2022.993289

**Published:** 2022-11-17

**Authors:** Han Cao, Xudong Hong, Heike Tost, Andreas Meyer-Lindenberg, Emanuel Schwarz

**Affiliations:** ^1^Department of Psychiatry and Psychotherapy, Central Institute of Mental Health, Medical Faculty Mannheim, Heidelberg University, Mannheim, Germany; ^2^Department of Computer Vision and Machine Learning, Max Planck Institute for Informatics, Saarbrücken, Germany; ^3^Department of Language Science and Technology, Saarland University, Saarbrücken, Germany

**Keywords:** machine learning, neuroscience, genetics, bioinformatics, biomarker, multi-task learning, multi-modal data analysis, translational research

## Abstract

Translational research in neuroscience is increasingly focusing on the analysis of multi-modal data, in order to account for the biological complexity of suspected disease mechanisms. Recent advances in machine learning have the potential to substantially advance such translational research through the simultaneous analysis of different data modalities. This review focuses on one of such approaches, the so-called “multi-task learning” (MTL), and describes its potential utility for multi-modal data analyses in neuroscience. We summarize the methodological development of MTL starting from conventional machine learning, and present several scenarios that appear particularly suitable for its application. For these scenarios, we highlight different types of MTL algorithms, discuss emerging technological adaptations, and provide a step-by-step guide for readers to apply the MTL approach in their own studies. With its ability to simultaneously analyze multiple data modalities, MTL may become an important element of the analytics repertoire used in future neuroscience research and beyond.

## Introduction

Many psychiatric disorders are thought to result from a complex interplay of genetic predisposition and exposure to environmental risk factors (1). As neuroimaging research has successfully identified brain-structural and functional alterations in illnesses such as schizophrenia, bipolar or major depressive disorder, there has been a strong interest in understanding how the illness’s genetic risk architectures contribute to such changes (2, 3). However, this risk architecture is complex and characterized by polygenicity and epistasis, with individual genetic polymorphisms explaining only little illness-associated variance. Similarly, there are significant hurdles in relating measures of peripheral gene expression to illness-associated differences in the brain. As a result, there is a substantial challenge in identifying a suitable strategy of how to investigate neurogenetic effects. Initially, a large body of literature from the “imaging genetics” field focused on testing associations between genetic variation in individual susceptibility genes and brain structure or function (2, 4). More recently, the increased availability of whole genome information and their aggregation into polygenic risk scores has fueled interest in testing associations between such scores and neural effects (4–6). These investigations reflect translational research strategies that are “sequential” in nature, i.e., they rely on the identification of illness-associated univariate or multivariate (e.g., polygenic) effects that are then tested for association with illness-relevant neural measures. Advances in the computational analysis of high-dimensional data have provided the opportunity for investigations in individual data modalities to become increasingly sophisticated. For example, deep learning is becoming a commonly applied tool for the exploration of neuroimaging data (7, 8) and is starting to be used for the analysis of genetics data (9). Such efforts will advance sequential translational research as more advanced computational approaches will explain larger portions of illness-relevant variation and thus lead to an improved understanding of how changes in different modalities interact to cause complex psychiatric phenotypes.

In this review, however, we would like to approach translational research from a different perspective. Advances in machine learning (ML) have created the possibility to identify sets of (e.g., biological) features that are simultaneously predictive of multiple outcomes. For example, it is now technically possible to identify a genetic signature that simultaneously predicts diagnostic grouping as well as illness-relevant brain function. This turns the classical, sequential approach of translation into a simultaneous exploration. The advantages are not only in the increased efficiency but, primarily, in the fact that it allows the identification of dimensions in high-dimensional data that may be of higher translational relevance. For example, while a conventional polygenic risk score may not predict illness-relevant differences in brain function, it is conceivable that there exists a polygenic measure predictive of both diagnosis and neural phenotypes, which cannot be identified from genetic association data alone. One machine learning approach facilitating such analyses is called multi-task learning (MTL) and has been successfully applied in numerous data-intensive fields, including biomedical informatics (10–14), speech and natural language processing (15, 16), image processing and computer vision (17, 18), as well as web-based applications (19, 20). Here, we present the MTL approach, describe its utility for neurogenetics analyses, and provide the reader with a step-by-step guide (see the Supplementary material) on how to apply this promising tool to his/her own data. The guide is based on the R package RMTL (21), which can be easily downloaded and installed from the CRAN website.^1^ As a methodological basis, the review will first focus on the so-called “regularization,” which reduces the complexity of machine learning models and builds a central element of MTL. Then, we describe the utility of widely applied as well as emerging MTL methods for neurogenetics analyses in psychiatry and provide an intuitive protocol for the application of common MTL approaches.

## Interpretable machine learning models for the exploration of neurogenetic effects

### Machine learning

**FIGURE 1:**
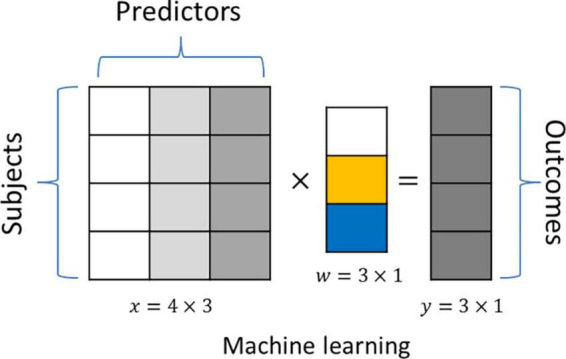
An example application of a linear machine learning model. The “outcome” *y* is predicted by the linear combination of three predictors (*x*), and we aim to identify the coefficient vector*w*.


An important goal of the machine learning method is to predict a given outcome by optimally combining multiple predictors in a linear (i.e., linear regression) or non-linear (i.e., random forest) fashion. These methods are called “supervised” because information about the outcome is available during the training phase of the model. Figure 1 shows an example of how linear machine learning predicts a given outcome y based on a linear combination of the input data *x* using coefficients *w*. In biomedical applications, the number of predictors frequently far exceeds the number of observations. This increases the risk of overfitting, where models fit too closely to a given training dataset and do not generalize well to unseen data. In addition to limiting predictive performance, such overfitting also obscures the true biological hallmarks underlying a given learning task, since these often show small effect sizes and are drowned out by stronger chance associations. This reduces the interpretability of the identified biological signatures due to an enrichment of false-positive predictors. A promising approach to address this is to integrate information related to the biological context of the prediction task into the machine-learning algorithm, i.e., *via* regularization.

$$\min_{w}ℒ\;\left(w|x,y\right)+\lambda \,\Omega \,\left(w\right)$$

Here, ℒ (*w*) refers to the loss function, which can be understood as a measure of “discrepancy” between the data distribution (*x*,*y*) and the built model. Minimizing this loss function over coefficients *w* leads to a model fit to a given dataset. Depending on the selection of the loss function, the machine learning method can be adapted to perform regression, classification or unsupervised tasks. For example, the least square loss ($ℒ\;\left(w\right)={||Y-X\,w||}_{2}^{2}$) is usually used for regression.

### Regularization

The function Ω(*w*) describes a “regularization” term, which is frequently also called the “penalty.” The function can be seen to penalize the discrepancy between the selected solution *w* and a set of assumptions made by the investigator. This strategy works well for high dimensional data, e.g., molecular “omics” data, because there usually exists an unlimited number of “optimal” solutions (that show similar predictive performance), and such strategy enables the algorithm to automatically select a more “interpretable” one among these.

This approach has been repeatedly applied in psychiatric research. In particular, regularization-based analysis for biomarker identification has been applied with a focus on various disorders, e.g., schizophrenia (22), bipolar disorder (23), and major depressive disorder (24). Compared to the conventional univariate analysis, a key factor contributing to the success of these approaches is that regularization-based machine learning allowed the unbiased integration of weakly outcome-associated signals distributed over the high-dimensional space (e.g., the entire genome) into a comparatively stronger risk score. The representative regularization approaches for this aim are the Lasso, ridge regression and elastic net (see Supplementary Table 1), which have already been applied to predict psychiatric phenotypes and explore genetic signatures associated with psychiatric illness (22, 25). In the Supplementary material, we detail how these approaches address challenges arising from high-dimensionality and strong correlation structures that are frequently presented in biological data.

Various regularization approaches have been proposed to identify biomarker candidates that are adapted to the specific structure of a given analysis question. We categorized these advanced methods into two classes: (I) penalization on the coefficients and (II) penalization on the difference between coefficients. A detailed explanation of these approaches can be found in the Supplementary material.

## Multi-task learning for multi-modal data analysis in neurogenetics

As the name suggests, the core principle of MTL is the attempt to simultaneously learn different prediction tasks (i.e., classification tasks). This aims to explore the underlying commonality between tasks, which may lead to improved generalizability and, potentially, more meaningful translational research. This review aims to highlight MTL’s utility for multi-modal data analysis. In biomedical applications, it is common to analyze the integration of heterogeneous but related data modalities, e.g., predictions at different time points during illness progression (26), case-control classification in different cohorts (27), or response prediction of multiple drugs (12). In psychiatric research, MTL has already been used to integrate schizophrenia markers from multiple cohorts (27) as well as measures of cognitive functioning and structural neuroimaging (28). By presenting MTL from different perspectives in the following sections, we hope to encourage the reader to identify useful applications in translational and neurogenetic research, such as the genetic prediction of illness risk and neural function (i.e., for identifying a genetic risk profile that shows neural effects) or the simultaneous prediction of diagnostic status and treatment response (i.e., to identify illness-associated biological patterns that are also responsive to treatment).

To enable knowledge transfer among tasks during the training, researchers exploit different methodological research lines. These research lines adopted different structures of variables to transfer the information, e.g., multi-task Gaussian processes shared covariance structure among tasks, and multi-task neural networks shared the hidden layers. Regularization-based MTL extended from regularized ML and has been among the most frequently employed methods due to its robustness and interpretability. Therefore, we provide here an introduction to “regularized MTL.” In the Supplementary material, we briefly summarize the current development of deep MTL due to its potential in neuroimaging studies.

### Multi-task learning

Regularized MTL builds upon the regularization strategies used for single-task machine learning. The penalty was designed to capture the multiple aspects of task-relatedness and can aid in increasing the generalizability as well as the interpretability of the models. This approach is usually formulated as:


$$\min_{w_{1},\ldots \,w_{i},\ldots \,w_{t}}\sum_{i=1}^{t}ℒ\;\left(w_{i}|X_{i},Y_{i}\right)+\lambda \,\Omega \,\left(w_{1},\ldots \,w_{i},\ldots \,w_{t}\right)$$

### Cross-task regularization

The regularization term Ω(*w*_1_, *w*_*i*_, *w*_*t*_) takes the coefficient vectors of all tasks as input and modulates relationships between the tasks according to the assumptions made by the investigators. From the perspective of penalization, the regularization term penalizes the degree of deviation between the learned models and such assumptions. λ controls the strength of the penalty. Thus by setting λ=0, the MTL models are “degenerated” to a set of single-task learning models. Usually, an optimal λ is selected *via* a resampling procedure, such as cross-validation.

### Multi-modal data analysis

In translational psychiatry, MTL is a promising approach because it allows the integrative analysis of clinically- and biologically relevant data modalities. Specifically, in multi-modal data analysis, MTL can differentiate information shared between and specific for different modalities (29). Unlike machine learning, which estimates a parameter for a predictor (e.g., the expression value of a gene) within a data modality, MTL estimates the parameters of biologically related predictors (e.g., the expression value of a gene and the DNA methylation at the methylation sites in chromosomal proximity to the same gene) across modalities simultaneously through regularization. This allows disentangling modality-shared effects from modality-specific effects.

### The intuition of categorization

**FIGURE 2:**
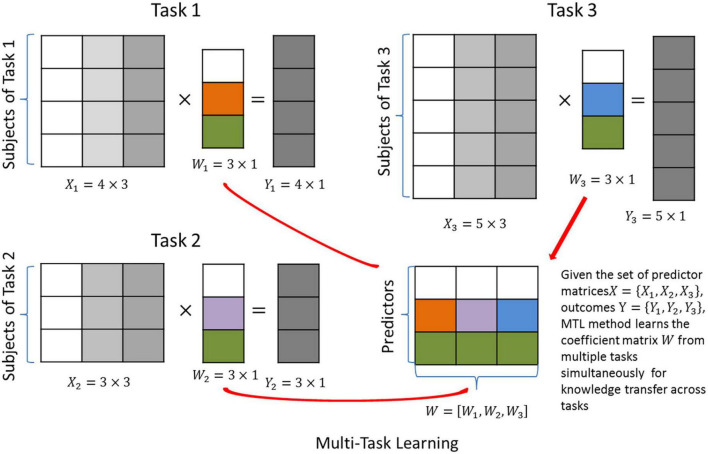
Graphical illustration of multi-task learning (MTL) with joint predictor selection. Three different tasks are predicted by the same set of predictors. The aim is to identify the coefficient matrix *W* that maximizes the prediction accuracy.

The regularization term Ω(*w*_1_,*w*_*i*_,*w*_*t*_) is frequently rewritten as Ω(*W*), where *W* = [*w*_1_,*w*_*i*_,*w*_*t*_]. Thus, predictors and tasks are indexed by the rows and columns of W, respectively (see Figure 2). Such a simplified form provides an intuitive interpretation of the cross-task regularization: it penalizes the complexity of the matrix *W* = *p*×*t*, and aims to identify a simple and representative structure of W that represents all tasks well. Thus, this form is also called “structural regularization” (30). Based on this interpretation, several regularization methods have been proposed. For example, in Figure 2, a row-wise sparse structure is explored for W, such that all three tasks are forced to share the same set of predictors.

Instead of structural regularization, other researchers described task-relatedness as a pairwise task similarity matrix or a network. Such methods can be understood as an extension of network-based single-task regularization (see Supplementary material), which penalizes the difference between tasks connected over a network and thus encourages the smoothness of coefficient vectors. Under this strategy, several studies have modeled real-world problems as MTL problems by engineering the assumed pairwise task similarities. Alternatively, instead of engineering the similarity matrix manually, another line of research has attempted to learn such a matrix from the data. The remainder of this section will describe methods and example applications falling into these two classes.

## Multi-task learning with structural regularization

Multi-task learning methods using structural regularization aim to identify a simplified structure of the coefficient matrix W, i.e., a sparse or “low-rank” structure. This means the coefficients to be learned can be represented in a compressed form that best describes the major variation in the data, leading to improved robustness against noise. As shown in Figure 2, an MTL method with joint predictor selection (31, 32) (see Supplementary Table 1) has been proposed as the multi-task version of the Lasso, which aims to select the predictors important to all tasks simultaneously.

This approach has been applied in the multi-omics analysis. For example, one study (33) integrated three gene expression and one genetic association datasets using this approach for predicting bone mineral density (BMD). This allowed the selection of genes and of biologically relevant SNPs that are simultaneously associated with BMD. Another work (34) adopted a similar approach to identify the shared imaging features that simultaneously predict two subtypes of bipolar disorders. The superior performance showed that the underlying biological heterogeneity of bipolar disorders could be disentangled by considering a detailed clinical characterization using MTL. Similarly, another work (35) identified the shared behavioral rhythms that simultaneously predict ten symptoms of schizophrenia. In other research fields, this method has been applied to overcome the problem of data scarcity in the transcriptomic analysis of cancer (36). The authors tested the approach on four breast cancer cohorts with different phenotypes, such as recurrence risk. By incorporating additional cohorts, the authors observed improved prediction accuracy for each task. Another study (37) applied the method successfully to integrate imaging-genetics data over multiple institutions to identify reproducible risk variants associated with Alzheimer’s disease (AD).

Another mainstream structural regularization approach is to explore the low-rank structure of the W matrix, the so-called “trace-norm” approach (see Supplementary Table 1). This method aims at identifying a shared low-dimensional space among all tasks and specific coefficients for each task. This method has been applied successfully to predict drug response by simultaneously exploring the associated molecular pattern of multiple drugs (12). *Via* the trace-norm regularization, multiple drugs (or coefficient vectors) were compressed in a low-dimensional space such that similar drugs (correlated coefficient vectors) were naturally clustered. The resulting low-rank modal strongly outperformed the single-task elastic net regarding prediction performance and biological plausibility. This method has not been applied to mental disorders to our knowledge. An example of a potential application could be comorbidity analysis with the objective to identify the, e.g., molecular signature that is simultaneously associated with multiple illness phenotypes. Such analysis would provide a low-dimensional representation of shared molecular effects and potentially provide insights into the biology of comorbidity.

## Multi-task learning incorporating pairwise task similarity

For MTL methods falling into this category, the task-relatedness is described as the pairwise task similarity matrix or the network. And the regularization penalizes the difference between tasks connected over the network. This method aims at identifying the coefficients that satisfy the shared similarity structure and explain the task-specific variation. A representative model in this category is the so-called “mean-regularized MTL” (38) (see Supplementary Table 1). Intuitively, the method assumes the existence of an identical latent model underlying all tasks, analogous to a “mean model” of all tasks. And the underlying true model is expected to be identified by considering the task-specific variations. This method has been used for the integration of multi-cohort gene expression datasets to identify expression signatures in brain samples from donors with schizophrenia (27). This study illustrated that MTL models showed higher reproducibility to unseen data cohorts than conventional machine learning methods and may thus be of particular use for the identification of reproducible biomarker signatures across studies.

By engineering the task-task similarity network according to assumed temporal relationships, MTL methods have further been applied to predict disease progression. For example, one study (26) identified MRI markers that track the progress of Alzheimer’s disease. The authors assumed the markers associated with disease progression change continuously and that the change of markers between adjacent time points is small. Consequently, a series of interpretable models was obtained by training the tasks simultaneously and penalizing the difference between two temporally adjacent tasks. In a follow-up study (14), several variations of the approach were proposed to introduce a sparse structure to the coefficient matrix. These variations aimed at selecting progression-related and task-specific predictors. We summarize these variations in Supplementary Table 1.

All these network-based regularization strategies can be unified in a framework by engineering a task similarity matrix with specific coefficients for a specific aim, e.g., modeling disease progression. A tutorial for designing the task similarity matrix was described elsewhere (39), and this feature is supported by the currently available MTL analysis software (21).

Instead of manually engineering the task similarity matrix, an interesting approach is to learn such a matrix from the data. One study (40) proposed such an approach, called “multi-task relationship learning” (40) (see Supplementary Table 1). Intuitively, this approach iteratively learns the model coefficients that fit the data and the task similarity matrix that fits the models. Another method (41) alternatively attempted to identify the clustering structure of tasks (called “convex clustered MTL”). This approach was derived from the spectral relaxation of the K-means clustering method. Notably, it was found (42) that the MTL with a low-rank structure also leads to a grouping of tasks, especially when there are large numbers of tasks. Due to its computational simplicity, the trace-norm model (see Supplementary Table 1) was suggested as the first approach for users to learn the similarity matrix of tasks. To our knowledge, these novel approaches have not been applied in psychiatry yet. One relevant problem these methods could address is the comorbidity and pleiotropy analysis of psychiatric disorders. These problems could comprise a large number of tasks (e.g., many potentially pleiotropic traits). This requires relatively large-scale data repositories for accurate estimation of the task similarity matrix. Such analyses may, for example, be useful for characterizing the genetic risk landscape of mental illness, and highlight risk contributions that are shared across, and specific to, different diagnoses.

## “Dirty” multi-task learning

The above MTL methods tend to work well when the task-relatedness is not confounded by unwanted biological and technical sources of variation. However, in biomedical data, such variations are common and can thus significantly impact MTL analyses. To make a reliable inference with MTL, it is essential to account for these unwanted variations, which gave rise to the development of so-called “robust” MTL methods. In this section, we described several regularization approaches to account for unwanted sources of variation during integrative data analysis, i.e., the inconsistent noise levels across tasks or outliers. Such “augmented” MTL methods may be suitable for real-world biological problems.

An interesting regularization method based on the “superposed structure” (43) originates from a class of statistical models called “dirty statistical models” (44), which assume that a given predictive pattern cannot be captured by any single model but can be interpreted well as a “superposition” of multiple base models (44). In the context of MTL, the “superposition” refers to a decomposition of the coefficient matrix into a sum of several independent matrices regularized by different methods (i.e., *W* = *P* + *Q*). The underlying rationale is to bolster MTL’s ability to capture the additional variation by incorporating additional regularization. These sources of variation are common in biological applications. For example, to select biological markers from multiple cohorts, the conventional MTL with joint feature selection (see Supplementary Table 1) naively assumes that all cohorts share the same predictive pattern and consistent noise levels across tasks. However, different cohorts might have originated from different institutions, potentially using different data acquisition protocols, leading to cross-cohort variability effects. Therefore, only exploring shared predictors may not maximize predictability across all tasks. One study (45) illustrated that the prediction performance of conventional MTL with joint feature selection was worse than that of the single-task elastic net if the extent of shared predictors is less than a given threshold or if the weights of the shared predictors are highly uneven. To address this issue, a dirty MTL (43) was proposed as a hybrid regularization (see Supplementary Table 1). This approach decomposes the regularization effect into one term capturing the shared predictors across tasks and one quantifying the association of the predictors with the individual task. This enables the algorithm to learn any extent of predictor sharing across tasks because the effects of individual tasks are isolated from the shared effect. Meanwhile, such a hybrid model avoids the loss of prediction accuracy due to the issue that predictors may not share representations across all tasks. A similar regularization strategy has been applied to predict depression severity using behavioral data (46). The data was collected from mobile devices, i.e., phones or wristbands, and used to predict the self-rated symptom severity score, as well as a clinical severity score. Here, the dirty model was used to capture the inconsistency between the self-reported and the clinical severity score. Another interesting dirty MTL method, called “robust MTL” (47), aims to detect outlier tasks. In addition to identifying the shared outcome-associated signature among tasks, another regularization component is used to remove outlier tasks, which are not sufficiently predictive or where the corresponding signatures do not overlap with the task-shared signatures. To our knowledge, this approach has not been applied in psychiatry yet. A potential example application is the integrative analysis of large-scale repositories. Since data quality, measurement techniques, sample size and cohort-specific properties may be heterogeneous across cohorts, it may be challenging to identify a single signature that predicts a given phenotype consistently across all repositories. The robust MTL has utility for this scenario because it allowes the identification of a signature set that is predictive of the target phenotype in most repositories and that can exclude outlying tasks.

## Emerging multi-task learning applications for multi-modal analysis in neuroscience

In this section, we will present two emerging areas in MTL research, which bear substantial promise for multi-modal analysis in neuroscience.

### High-order multi-task learning

**FIGURE 3:**
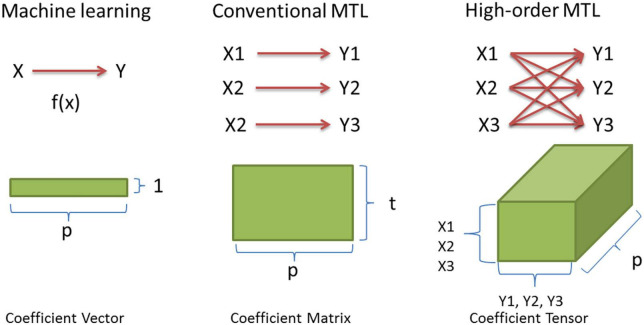
From machine learning (ML) to High-order MTL. In ML, one functional mapping links the predictors and the outcome, and the coefficients are represented as a vector. In conventional MTL, similar to ML, multiple “one-to-one” mappings link the predictor and outcome domain, where the mappings are not crossed among tasks. The resulting coefficients are represented as a matrix. In high-order MTL, highly complex multi-modality is accounted for by “multiple-to-multiple” mappings to connect the predictor with the outcome domain. The coefficients are represented as a tensor.

There is an emerging trend in the MTL community to represent the predictor coefficients in the form of a tensor (i.e., a 3-dimensional matrix) aiming to represent the complex relationships between multiple data modalities. We denote this type of method as “high-order MTL.” Figure 3 illustrates the evolutional path from conventional machine learning to high-order MTL, as well as the difference between high-order and conventional MTL. In many real applications, multiple data modalities (X1∼X3 in Figure 3) and multiple outcomes (Y1∼Y3 in Figure 3) are available, where any single outcome can be predicted by any data modality with a certain level of accuracy. This task relationship cannot be captured appropriately by conventional cross-task regularizations because the data modalities in conventional MTL are not cross-mapped to the outcomes. High-order MTL offers a potential solution for this increasingly common scenario. One study (48) proposed a high-order MTL formulation, called “multilinear MTL” by employing the tensor-based trace-norm regularization (see Supplementary Table 1) to identify the shared pattern across tasks. The authors demonstrated the superior performance of the proposed method compared to conventional MTL using internet data. This method appears suitable for modeling the genetic and molecular basis of psychiatric and neuroimaging phenotypes. With high-throughput technologies, different data modalities, including gene expression and genetic association data, can be analyzed simultaneously. Any of these data modalities can be associated with any given brain-imaging measure with a certain level of accuracy. Such “multiple-to-multiple” mappings are ideal applications of high-order MTL.

Another high-order MTL formulation aims to learn a consistent pattern of predictor interactions across tasks and is called “multi-task predictor interaction learning (49).” This formulation adds pairwise predictor interactions in the regression as a matrix of variables (see Supplementary Table 1). Then by stacking such matrices across tasks, the interaction tensor is regularized by introducing the sparse and low-rank structures. We summarize the formulations in Supplementary Table 1. The authors demonstrated the superior performance of the method using educational data. This method may also have significant utility for capturing biological interactions such as those present in co-regulated biological networks and biological pathways relevant to psychiatric disorders such as schizophrenia (50). In machine-learning analysis, studies have incorporated co-expression information to improve gene marker discovery (51), but few have tested the predictability of the gene interactions for diagnosis. This may be due to the large number of coefficients that require estimation, limiting the statistical power. The corresponding MTL approaches offer the possibility to improve statistical power by incorporating additional samples and constraining model complexity with regularization.

### Distributed multi-task learning

**FIGURE 4:**
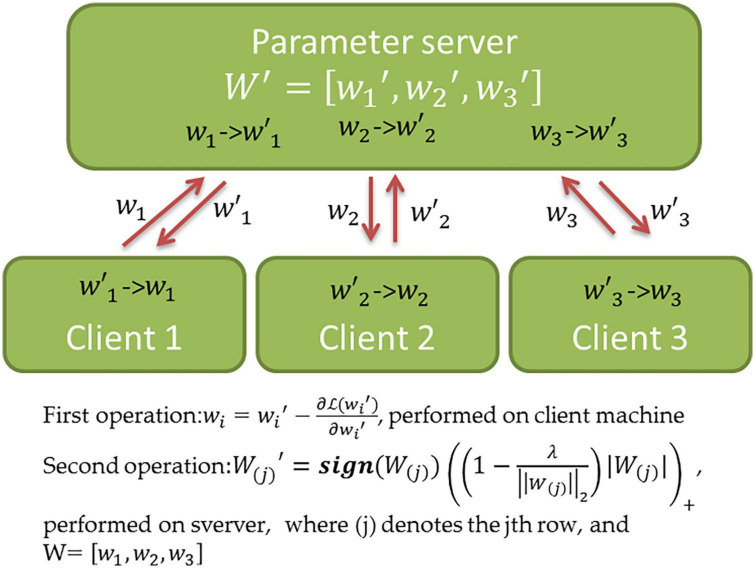
Schematic overview of distributed MTL. The Figure shows the distributed computations and parameter exchange of the MTL method with joint predictor selection (see Supplementary Table 1 for details). Once the algorithm starts, the client *i* performs a one-iteration gradient descent and transfers the result*w_i_* to the server. After the server collected messages from all clients, the second operation is performed, and the updated $w_{i}^{\prime }$ is returned to client *i*. This procedure is repeated until the convergence of the solution (*W*′≈*W*).

It is straightforward to parallelize the training of MTL models, which facilitates their distributed application on geographically distributed data sources. This is because the optimization procedure of most regularization-based MTL methods can be disentangled into two operations performed independently. As shown in Figure 4, the first operation entails identifying a potential solution for fitting the data on the local client. The second operation performs the cross-task knowledge transfer on a central server. Two operations are performed iteratively. Therefore, the first operation can be easily parallelized. Figure 4 demonstrates a simple distributed strategy for MTL with joint predictor selection (see Supplementary Table 1), which may not be efficient in practice. For example, the parameter server only performs the second operation after collecting all messages from the clients, and thus, the computing speed is limited by the slowest network connection. To address this, one study (52) proposed an asynchronous approach for distributed MTL such that the first operation need not wait for the response from the server. This results in a substantially improved efficiency but an earlier convergence. In recent years, many conventional MTL methods have been adapted to distributed learning, i.e., the distributed multi-task kernel machine (53) and distributed MTL with network incorporation (53).

Current genetic and neuroimaging research substantially profits from the establishment of large consortia involving numerous institutions, e.g., IMAGEMEND (54) and ENIGMA (55). The collaborative network ENIGMA comprises over 200 research institutions from 43 countries. In such a scenario, the protection of data privacy, as well as the logistics of data analysis across institutions, becomes challenging. Distributed MTL offers the possibility to naturally integrate data resources across hospitals into a machine-learning framework. To prevent the data leakage of patients during the integration procedure, a new research line emerged called “federated machine learning” (56), which aims at training the distributed model in a privacy-preserving fashion. The security and privacy issues relevant to federated learning systems are summarized in Box 1. The techniques for addressing security issues were classified as “proactive” and “reactive” techniques. The former type progressively guesses the potential risks of the system and performs a defensive procedure, whereas the latter type initiates after the attacks occurred. Blockchain (57) has been applied in a federated learning system to support both defensive techniques. Blockchain provides an “immutable ledger” for saving the contributions of each node to the global model such that the malicious change of the global model can be detected before or after the attacks. For privacy issues, researchers introduced cryptologic algorithms into the distributed learning framework, in order to prevent the leakage of individually identifiable information. Differential privacy (58) is one of such algorithms, and aims at statistically erasing identifiable characteristics from a well-trained model. More countermeasures for these challenges have been explored in Mothukuri et al. (59). There are already studies adopting this methodology to perform data integration across hospitals. For example, one study integrated COVID-19 patients’ information across multiple hospitals for training a prediction model using a variant of a federated algorithm (60). Recently, a user-friendly federated MTL software supporting multi-modal biomedical analysis has been developed (29).

**Security and privacy issues of federated learning.**

Federated learning (FL) is a new and fast-growing area. Questions related to the security and privacy of information are of particular relevance for FL approaches and subjects of active research.

Security refers to the vulnerability of FL systems that could be abused by a potential attacker with the aim to break the system (59). For example, the FL systems require substantial communication between nodes in order to update a given model, which is associated with significant risks; in the client-server architecture, the central server is an important node connecting all clients, and may become the target of an attack. A common type of such an attack in the context of security is the so-called “poisoning”. In this attack, the malicious client node updates the local model with malicious data points or parameters, causing the global model to lose its predictive capacity (83).

Privacy issues refer to the threats that can reveal a given individual’s sensitive information by exploring the models’ parameters (59). This type of attacks are also termed “inference attacks”. For example, membership inference attack aims at identifying whether certain data were used as part of the training set.

Another application scenario of federated MTL with relevance to psychiatry is the analysis of mobile data collected by handheld and wearable devices. In recent years, the widespread distribution of these devices enabled the monitoring of emotional and cognitive changes in the daily life context and for intervention tailored by personalized and context-specific information (61). These daily life data allowed investigating, e.g., psychosis in daily life, which might improve our understanding of this condition and lead to a better mechanistic model (61). One study (62) already showed improved prediction accuracy (82%) using ESM (experience sampling method) data collected from mobile devices. Federated MTL could have useful application scenarios in this research field. First, the heterogeneity of mobile data collection could be modeled by federated MTL. Mobile devices usually contain many sensors and recording channels, thus generating multi-modal data. Modeling such cross-modality heterogeneity could provide an opportunity for investigating psychiatric outcomes more comprehensively. In fact, a recent study (63) described a multi-task dynamic system for analyzing multi-modal time series, which built the theoretical and algorithmic framework for solving this problem. Second, the temporal dependency of mobile data could be modeled by federated MTL. Mobile data usually represents time series violating the I.I.D assumption, and MTL could capture the temporal relationship by cross-task regularization. A similar concept has been pursued to model disease progression (14).

## Discussion

In this review, we provide a comprehensive overview of MTL methods that have the potential to substantially advance translational research in neuroscience. MTL is an emerging technology that has already been applied extensively in biomedical studies and, occasionally, in neuroscience. With its ability to account for complex relationships across different data modalities, MTL appears suitable for integrative, multi-modal data analyses in neuroscience. As a data integration tool, MTL can provide an increase in statistical power, due to the assumpted cross-task structure and the resulting compression of the search space of model parameters.

An interesting property of MTL with regard to its application in translational neuroscience is that it allows for a “parallelization” of the identification of biological (and other) signatures and may thus accelerate the discovery of clinically useful and mechanistically informative patterns. We argued in this review that this approach might be particularly useful for the analysis of multi-modal data. To illustrate this, we first explored the utility of regularization-based machine learning methods with regard to high data dimensionality and complex feature correlation, and categorized these methods according to specific use cases relevant to neurogenetics studies. We then introduced the MTL concept and categorized methods according to specific methodological characteristics for capturing the task relationships, with possible applications for the integration of multiple data modalities. To illustrate the technical properties of these methods, we provided information on relevant considerations in the Supplementary material. Finally, we highlighted two emerging areas in MTL that might be of benefit for future studies in neuroscience. With the increasing richness of available data resources, capturing the relationships between data modalities will be a promising but challenging focus of machine learning analyses. As the more complex “multiple to multiple” relationship among tasks is beyond the capacity of conventional MTL, high-order MTL may offer a potential solution. The utilization of large-scale data resources is further aided by approaches such as federated machine learning, which does not require data to be brought together into a single storage solution. This will support the integrative analysis of sensitive data, as well as of data that cannot be directly shared due to their large scale. While approaches such as regularized MTL are particularly effective for high-dimensional data analysis, in low-dimensional scenarios, there are similar approaches in the regression analysis framework that have utility for multi-modal data analysis. Approaches of interest in this context include multilevel regression (64) and multivariant regression (65, 66).

In translational psychiatry, multi-modal machine learning approaches have already been applied in numerous studies, including for personalized medicine approaches. Some of such studies support that clinical decision-making can potentially be improved through AI-based integration of information from other data modalities. In this context, MTL may be a valuable addition to the repertoire of available computational techniques, due to its ability to simultaneously link multiple, clinically relevant phenotypes. Interesting application areas could include the integration of audio, video and text-based data, as has been previously investigated for improving the diagnostic classification of bipolar disorder and depression (67). Another similar example is the integration of audio and text data from social media platforms (68), and more studies have been reviewed in (69).

Another interesting example is the integration of multimodal data for the development of prognostic models, e.g., using clinical and neuroimaging data for the prediction of social functioning in high-risk psychosis (83% accuracy) or recent onset depression patients (70% accuracy) (70). These accuracy estimates were higher than those obtained from human experts, highlighting the possible utility of computer-aided prognostic models. Another prognostic study (71) successfully predicted the transition to psychosis by integrating clinical, neurocognitive, neuroimaging, and genetic information. These analyses support the utility of multimodal integration for the development of machine learning models in psychiatry, and point to possibilities for the application of MTL that may further refine such approaches. Such analyses may be particularly relevant for the integrative analysis of distributed, large-scale data repositories, where MTL could offer a computational approach for disentangling sources of heterogeneity that may affect the cross-repository generalizability of models (72, 73). In this context, federated applications of MTL are of particular relevance, and we here presented several methodological considerations.

Beyond the predictive accuracy and the generalizability of models, MTL may have substantial utility for improving our mechanistic understanding of mental illness by linking multiple data modalities in a single analysis. Interesting examples include the special correlation between brain-structural and –functional effects (74) that may support a mechanistic relevance for illnesses such as schizophrenia (75). Multimodal integration of brain alterations has been performed repeatedly, using techniques such as canonical correlation analysis (76), deep learning (77), and other methodologies (78), which may be well complemented by MTL strategies. A particularly interesting focus area would be the integrative analysis of resting state and task-based MRI in relation to cognitive functioning relevant to mental illness (79, 80). Linking these modalities directly through MTL could provide a deeper insight into mechanisms involved in the process underlying potentially transdiagnostic symptom clusters, and thus contribute to a dissection of patient heterogeneity through a more in-depth understanding of the underlying neurobiology. By identifying shared mechanistic effects across data modalities, multitask learning may thus aid in stratification that has a direct connection to a clinically relevant phenotype, which may aid in overcoming typical challenges associated with unsupervised clustering tools. The relevance of multitask learning for such integrative analysis is supported by emerging studies that, for example, focus on the integrated analysis of neuroimaging and genetic association data for prediction of schizophrenia (81) or of the disease stages of Alzheimer’s disease (82). With the ever-increasing availability of large-scale, multi-modal data, we believe these emerging algorithms will find innovative applications in translational psychiatric research.

## Conclusion

Multi-task learning approaches offer a computational framework for multi-modal data analysis, and could allow dissecting data heterogeneity. MTL has numerous interesting application scenarios in biomedical research, including patient stratification, comorbidity modeling, multimodal data integration, disease process modeling, or multi-cohort biomarker discovery. We thus believe that it will be increasingly applied in psychiatric research, could contribute to an improved mechanistic understanding of mental illness, and may provide the basis for novel, clinically useful applications.

Security refers to the vulnerability of FL systems that could be abused by a potential attacker with the aim to break the system (59). For example, the FL systems require substantial communication between nodes in order to update a given model, which is associated with significant risks; in the client-server architecture, the central server is an important node connecting all clients, and may become the target of an attack. A common type of such an attack in the context of security is the so-called “poisoning.” In this attack, the malicious client node updates the local model with malicious data points or parameters, causing the global model to lose its predictive capacity (83).

## Author contributions

HC conceived, designed, and wrote the manuscript. XH contributed to the deep learning section. ES wrote and revised the manuscript. HT and AM-L revised and commented on the manuscript. All authors contributed to the article and approved the submitted version.

## Footnotes

1
https://cran.r-project.org/
