# Supplementary material for: Advancing translational research in neuroscience through multi-task learning
Source: Front Psychiatry. 2022 Nov 17;13:993289. doi: 10.3389/fpsyt.2022.993289 (PMC9714033; doi:10.3389/fpsyt.2022.993289)
Supplement: Supplementary file 1 [file Data_Sheet_1.docx]

| Regularization Name | Type | Subtype | Math Form | Effect | Ref |
| --- | --- | --- | --- | --- | --- |
| Lasso | Machine Learning | Penalization on coefficients | $\Omega(w)=\lambda{\vert\vert w\vert\vert}_{1}$ | Remove irrelevant predictors | ^1^ |
| Ridge Regression |  |  | $\Omega(w)=\lambda{\vert\vert w\vert\vert}_{2}^{2}$ | Prevent overfitting | ^2^ |
| Elastic net |  |  | $\Omega(w)=\lambda(\alpha{\vert\vert w\vert\vert}_{1}+\frac{(1-\alpha)}{2}{\vert\vert w\vert\vert}_{2}^{2}$ | Select important predictors with grouping effect | ^3^ |
| Group Lasso |  |  | $\Omega(w)=\lambda\sum_{g=1}^{G} {\vert\vert w_{I_{g}}\vert\vert}_{2}$ | Account for group structure over predictors | ^4^ |
| Sparse Group Lasso |  |  | $\Omega(w)=\lambda_{1}{\vert\vert w\vert\vert}_{1}+\lambda_{2}\sum_{g=1}^{G} {\vert\vert w_{I_{g}}\vert\vert}_{2}$ | Account for sparse group structure over predictors | ^5^ |
| Fused Lasso |  | Penalization on the difference between coefficients | $\Omega\left( w \right)={\lambda_{1}\vert\vert w\vert\vert}_{1}+\lambda_{2}\sum_{i=1}^{p} \vert w_{i}-w_{i-1}\vert$ | Account for ordered predictors | ^6^ |
| Absolute Fused Lasso |  |  | $\Omega\left( w \right)={\lambda_{1}\vert\vert w\vert\vert}_{1}+\lambda_{2}\sum_{i=1}^{p} \vert\vert w_{i}\vert-\vert w_{i-1}\vert\vert$ | Account for ordered predictors (only magnitudes matter) | ^7^ |
| Network-based Regularization |  |  | $\Omega\left( w \right)={\lambda_{1}\vert\vert w\vert\vert}_{1}+\lambda_{2}w^{T}Lw$  $\Omega\left( w \right)={\lambda_{1}\vert\vert w\vert\vert}_{1}+\lambda_{2}\sum_{(i,j)\in E} \max_{w_{i}, w_{j}} \left\{ \left\vert w_{i} \right\vert, \vert w_{j}\vert\right\}$ | Incorporate network structure over predictors | ^8,9^ |
| Joint Feature Selection | Multi-Task Learning | MTL with structural regularization | $\Omega\left( W \right)={\lambda\vert\vert W\vert\vert}_{2,1}$ | Select predictors important to all tasks simultaneously | ^10^ |
| Trace-norm model |  |  | $\Omega\left( W \right)={\lambda\vert\vert W\vert\vert}_{*}$ | Find the low-rank structure of the models | ^11^ |
| Mean-regularized model |  | MTL incorporating pairwise task similarity | $\Omega\left( w_{1},\ldots w_{i}, \ldots w_{t} \right)=\lambda\sum_{i=1}^{t} {\vert\vert w_{i}-\frac{1}{t}\sum_{j=1}^{t} w_{j}\vert\vert}_{2}^{2}$ | Identify the mean model as the latent model behind all tasks | ^12^ |
| Temporal Smoothness Prior |  |  | $\Omega\left( w_{1},\ldots w_{i}, \ldots w_{t} \right)=\lambda_{1}\sum_{i=1}^{t-1} {\vert\vert w_{i}-w_{i+1}\vert\vert}_{2}^{2}+\lambda_{2}{\vert\vert W\vert\vert}_{2}^{2}$  $\Omega\left( w_{1},\ldots w_{i}, \ldots w_{t} \right)=\lambda_{1}\sum_{i=1}^{t-1} {\vert\vert w_{i}-w_{i+1}\vert\vert}_{2}^{2}+\lambda_{2}{\vert\vert W\vert\vert}_{2,1}$  $\Omega\left( w_{1},\ldots w_{i}, \ldots w_{t} \right)=\lambda_{1}\sum_{i=1}^{t-1} {\vert\vert w_{i}-w_{i+1}\vert\vert}_{1}+\lambda_{2}{\vert\vert W\vert\vert}_{2,1}+\lambda_{3}{\vert\vert W\vert\vert}_{1}$ | Incorporate temporal order among tasks to predict the disease progression | ^13,14^ |
| Multi-task relationship learning |  |  | $\Omega\left( W, S \right)=\frac{\lambda_{1}}{2}tr\left( WW^{T} \right)+\frac{\lambda_{2}}{2}tr\left( WS^{-1}W^{T} \right)$  s.t. $\left\{ S≽0, tr\left( S \right)=1 \right\}$ | Learn task-relationship | ^15,16^ |
| Convex Clustered MTL |  |  | $\Omega\left( W,M \right)=\lambda\alpha\eta(1+\eta)tr(W{(\eta I+M)}^{-1}W^{T})$  s.t. $\left\{ tr\left( M \right)=k, M≼1, M\in S_{+}^{m} \right\}$ | Incorporate clustered structure between tasks | ^17,18^ |
| MTL with dirty model |  | Accounting for biological variations | $\Omega\left( W \right)={\lambda_{1}\vert\vert P\vert\vert}_{\infty,1}+\lambda_{2}{\vert\vert Q\vert\vert}_{1}$ s.t. $W=P+Q$ | Identify the shared and task-specific predictors simultaneously | ^19^ |
| Robust MTL |  |  | $\Omega\left( W \right)={\vert\vert P\vert\vert}_{2,1}+{\vert\vert Q^{T}\vert\vert}_{2,1}$ s.t. $W=P+Q$ | Detect the outlier tasks | ^20^ |
| Multilinear MTL | High-order MTL |  | $\Omega\left( \mathcal{W} \right)={\lambda\vert\vert\mathcal{W\vert\vert}}_{*}\mathcal{, W=}p\times t_{1}\times t_{2}$ | Model the complex task-relationships | ^21^ |
| Multi-task predictor interaction learning |  |  | $\Omega\left( \mathcal{Q} \right)=\lambda\sum_{i=1}^{p} \sum_{j=1}^{p} \sqrt{\sum_{k=1}^{t} (\mathcal{Q}_{ijk}^{2}+\mathcal{Q}_{jk}^{2})}$  $\Omega\left( \mathcal{Q} \right)=\lambda\sum_{i,j} \sqrt{\sum_{k=1}^{t} \mathcal{Q}_{ijk}^{2}}$  $\Omega\left( \mathcal{Q} \right)=\lambda\mathcal{\vert\vert Q\vert\vert}_{*}\mathcal{, Q=}p\times p\times t$ | Identify the simple and representative structure of predictor interaction patterns across tasks | ^22^ |

**Supplementary Table 1.** Variations of regularization strategies for machine learning, multi-task learning and high-order multi-task learning.

**Algorithmic implementation of MTL**

To solve these approaches listed in Supplementary Table 1 efficiently, researchers usually model the regularization term $\Omega\left( \mathcal{W} \right)$ in the convex form or a complex form which allows a decomposition into a series of simpler convex forms due to the well-studied complexity bound of the convex function. For example, $\Omega\left( W \right)={\lambda||W||}_{2,1}$ is the convex form for achieving the joint feature selection. To identify the low-rank structure of the coefficient matrix, $\Omega\left( \mathcal{W} \right)=rank(W)$ is ideal for such a purpose. However, due to its non-convexity, this form is computationally intractable and usually relaxed to the trace-norm approach. For the multi-task relationship learning approach, the formulation $\Omega\left( W, S \right)=\frac{\lambda_{1}}{2}tr\left( WW^{T} \right)+\frac{\lambda_{2}}{2}tr\left( WS^{-1}W^{T} \right)$ is not convex and can be solved with the alternative gradient approach due to its piecewise convex nature. First, when S is fixed, the regularization term becomes $\Omega\left( W \right)=\frac{\lambda_{1}}{2}tr\left( WW^{T} \right)+\frac{\lambda_{2}}{2}tr\left( WS^{-1}W^{T} \right)$, which aims to incorporate the task relationship to regularize W. Second, when W is fixed, the regularization term becomes $\Omega\left( S \right)=\frac{\lambda_{2}}{2}tr\left( WS^{-1}W^{T} \right),$s.t. $\left\{ S≽0, tr\left( S \right)=1 \right\}$ , which aims to identify a simple structure of S while retaining the task relationship. During the optimization procedure, W and S are optimized alternatingly until convergence.

For convex MTL approaches (which applies to most methods shown in supplementary table 1), the state-of-art solver is to apply the so-called “proximal operator”^23^, which analyzes the non-smooth penalty using a high-order approximation method. This method can then be accelerated by the momentum method, e.g., Nesterov’s accelerated method^11,24^. Furthermore, to learn the sparse MTL approaches, a training procedure with a warm start is usually applied to learn a stable sparsity.

**Technical considerations of lasso, ridge and elastic net for biomedical applications**

Lasso, ridge and elastic net have been widely applied for data analysis in psychiatry, and are robust against high data dimensionality and complex correlation structures.

**High dimensionality** With regards to high data dimensionality, the lasso, for example, aims to find a solution $w$ with a low number of predictors, meaning that most coefficients of $w$ are penalized to 0. In practice, such a method is usually seen as a combined procedure of model fitting and predictor selection. One study ^25^ applied the lasso to explore the genetic underpinnings of brain structure using MRI data. The authors successfully identified a small set of SNPs harbored by 22 genes, which were significantly associated with temporal lobe volume. Lasso works well when many outcome-irrelevant predictors exist, such that these can be ‘filtered out’ automatically by the lasso, while improving predictive performance. These properties make the Lasso method suitable for high-dimensional data analysis.

**Complex correlation** Biological predictors can usually be grouped into meaningful sets that may, in turn, be exploited for machine learning. First, there may be strong statistical correlations between predictors. For example, the expression levels of co-expressed genes are correlated, and such genes are frequently involved in the same biological processes. Similarly, genetic variants in chromosomal proximity tend to be correlated due to LD. Second, even in the absence of statistical correlation, biological ontologies, such as the GeneOntology^26^, allow the categorization of biological predictors depending on their biological function, molecular mechanisms or subcellular localization. Such summarization enhances the biological interpretation of identified gene sets by identifying the predominantly involved biological themes^27^. Third, some applications categorize biological predictors according to spatial or temporal effects. For example, in longitudinal studies exploring the progression of illnesses such as Alzheimer’s disease, biological predictors may be selected given their temporal correlation. Spatial correlations are, for example, routinely exploited in MRI studies where structural and functional effects are correlated depending on the spatial context of the brain. These scenarios of grouping effects have been analyzed statistically in the Lasso studies^3^. When grouping effects occur (e.g., multiple predictors show similar behavior across subjects), lasso does not select all of these predictors but tends to select a single representative. In this scenario, the ‘elastic net’ algorithm has desirable properties, as it combines the penalty terms of the lasso and the so-called ‘ridge regression’, using the extra penalty ($l_{2}$-norm) term to select sets of correlated predictors.

**The categorization of methods for regularized machine learning**

Penalization on coefficients

The so-called ‘group Lasso’ has been applied for phenotype prediction^28^, biomarker discovery^5^ and the investigation of genetic associations ^29^. This method considers the grouping structure across predictors and penalizes at such group level to arrive at a “sparse” solution, i.e., where coefficients of unimportant groups are set to zero. As a result, unimportant groups of predictors are filtered out while the generalization ability of the algorithm is maximized. The penalty takes the form$\Omega(w)=\lambda\sum_{g=1}^{G} {||w_{I_{g}}||}_{2}$, where $G$ represents a set of groups. In one study^28^, aiming to identify transcriptomic markers of cancer, the authors applied the group lasso penalty to select the most significant gene clusters. The clusters were determined using K-means clustering before machine learning. This resulted in the identification of 20 to 60 biologically plausible genes, selected from 500 candidates. One disadvantage of this method is that it naively assumes the absence of a sparse pattern within a given predictor group. The assumption is too strong for many applications. Therefore, in previous work^5^ the $l_{1}$-norm was combined with the group lasso to allow for within-group sparsity, and this technique has been termed ‘sparse group lasso’ with penalty term $\Omega(w)=\lambda_{1}{||w||}_{1}+\lambda_{2}\sum_{g=1}^{G} {||w_{I_{g}}||}_{2}$. One successful application of this approach has been an unsupervised analysis across omics modalities ^30^. This study explored the association between genetic and transcriptomic predictors using canonical correlation analysis. By introducing the sparse group structure into the model, the authors demonstrated a higher true positive rate than obtained using conventional methods.

Penalization on the difference of coefficients

Another extension to the Lasso method is to penalize the difference between coefficients. The primary approach is the so-called ‘fused Lasso’ ^6^ ($\Omega\left( w \right)={\lambda_{1}||w||}_{1}+\lambda_{2}\sum_{i=1}^{p} |w_{i}-w_{i=1}|$), which assumes the predictors to be ordered and the difference between adjacent predictors to be small. This penalty encourages the smoothness of coefficients across predictors while retaining the maximum predictability. This strategy has been applied successfully to GWAS studies, to account for the LD effects ^7,31^. One study ^31^ compared the fused Lasso method to conventional lasso and linear regression in genetic data from patients with rheumatoid arthritis and highlighted the improved ability to select risk variants.

An interesting application area for such approaches is the incorporation of biological network information into the machine learning procedure. For this, an extension of the fused lasso, called ‘network-based regularization’, was proposed (i.e., $\Omega\left( w \right)={\lambda_{1}||w||}_{1}+\lambda_{2}w^{T}Lw$, where $L$ is the graph Laplacian). This strategy penalizes the difference between two predictors connected in the network and thus encourages the coefficients’ smoothness over the network. Such methods have been repeatedly applied to incorporate information from different kinds of biological networks^32^, including protein-protein interaction^33^ and co-expression^34^ networks. To obtain a more comprehensive understanding of the network-based analysis, readers are referred to a review^32^. In an interesting application^34^, transcriptomic biomarkers of cancer were explored by incorporating the network structure obtained from KEGG pathways, leading to the identification of a gene network that demonstrated higher biological plausibility than gene sets derived from conventional lasso.

A weakness of the fused lasso and network-based regularization is that the coefficients of negatively correlated predictors can be incorrectly regularized by these methods. For example, considering two inversely co-regulated genes, where one gene is positively correlated with the outcome and the other is inversely correlated, the sign of the coefficients should be inverted relative to each other. However, the above regularization strategies encourage the signs of the coefficients to be the same, which may bias the result. A study^7^ analyzed such a scenario and proposed an improved version of the fused lasso, called ‘absolute fused lasso’ (AFL). The corresponding penalty ($\Omega\left( w \right)={\lambda_{1}||w||}_{1}+\lambda_{2}\sum_{i=1}^{p} ||w_{i}|-|w_{i-1}||$) penalizes the difference between the absolute values of the coefficients instead of the actual coefficients and thus encourages the smoothness of negatively co-regulated predictors. Training this algorithm is challenging since this penalty is non-convex. Therefore, the authors proposed to address the local optima by DC (difference of convex functions) programming^35^. The algorithm reduced the non-convex optimization problem to a series of convex problems. The method was tested on genetic association data, aiming to identify expression quantitative trait loci (eQTL) and achieved a higher prediction accuracy and true positive rate than conventional methods.

Similar issues have been raised for network-based regularization and addressed using different techniques^34,36^. For example, in one study^10^, the signs of two coefficients connected via a network were estimated before the machine-learning stage. Then the algorithm utilized the estimated signs for training. The signs were estimated by quantifying the correlation between the predictor and the outcome. Based on the algorithm, the transcriptomic profile associated with the biological response to infection and vaccination was explored, and improved interpretability of results was found when embedding 10 biological networks sequentially. Particularly, the authors found that incorporating different types of biological knowledge produces a model capturing distinct aspects of the underlying biology. Instead of directly estimating signs, other researchers found a convex alternative formulation for replacing the non-convex part^8,9^, for example,$\Omega\left( w \right)={\lambda_{1}||w||}_{1}+\lambda_{2}\sum_{(i,j)\in E} \max_{w_{i}, w_{j}} \left\{ \left| w_{i} \right|, |w_{j}| \right\}$. Such min-max term convexly penalizes the larger absolute value of two given coefficients, and thus reduces the difference between the magnitudes of the two coefficients.

This section presented regularization methods that are fundamental components of many conventional machine learning methods and also a central part of MTL. The next section will show how these techniques are extended in MTL, to provide a basis for exploring the utility of MTL for translational medicine and neurogenetics analyses.

More specifically, the shared predictors are captured by the row-wise sparse structure (P), and the task-specific predictors fall into the element-wise sparse structure (Q).

**Deep MTL**

While MTL has already been applied successfully to analyze numerous high-dimensional classification and prediction tasks, its predictive capacity in some modalities, such as neuroimaging or DNA sequence data, may profit from further methodological innovation. For these modalities, tremendous progress has been made in the field of deep learning. While conventional machine learning mostly relies heavily on the so-called ‘feature engineering’, i.e. the utilization of domain-expertise to turn raw data into useful input predictors for machine learning algorithms, methods such as deep neural networks (DNN) frequently aim to identify predictive signals from a large-scale raw data, learning hierarchies of increasingly complex predictor representations. The extension of the deep learning methodology towards multi-task learning is a promising avenue for combining the strengths of both methodological approaches. Therefore, in the last methodology section of this article, we briefly introduce DNN-based MTL.

Starting from the 2000s, DNNs achieved superior prediction performance in many fields including computer vision^37^, speech recognition^38^, natural language processing^39^ and computational biology^40^. In psychiatry, using neural imaging data, DNNs have successfully been applied for diagnostic classification in Alzheimer’s disease^41^, ADHD^42^ and schizophrenia^43^, with most studies reporting accuracies exceeding 90%. For a comprehensive review of DNN applications in psychiatry, readers are referred to Durstewitz et al. ^44^. The reasons for the success of DNNs have not been understood completely. In part, a DNN provides a large-enough space of “computable functions” using a relatively small number of parameters, which is achieved by stacking parameters layer by layer. As a result, DNNs can learn better representations of complex patterns from the data but still require large training data to prevent overfitting.

Here, we specifically focus on a deep learning application that simultaneously learns prediction tasks in multiple modalities. This approach is denoted here as ‘deep MTL’. Similar to conventional MTL, the key element of deep MTL is the mechanism of knowledge transfer across tasks^45^. One strategy, called “soft sharing”, adopts the methodology of conventional cross-task regularization. By penalizing the dissimilarity and sharing the commonality across two or multiple neural networks (i.e., hidden parameters), the assumption of task-relatedness is incorporated. However, the generalization ability highly depends on the accuracy of the assumed task-relatedness, which may be difficult to design in practice. Alternatively, another strategy, called “hard sharing”, requires fewer prior assumptions, and has been more widely used for deep learning. Instead of focusing on the commonality across neural networks, this strategy directly shares the hidden layers across tasks. The underlying principle is the recognition of the roles of the different layers. For example, for analysis of imaging data, the low-level layers (closer to the input) tend to recognize pixel-related predictors, i.e., points or lines, while the high-level layers (closer to the output) tend to capture abstract predictors, i.e., shapes, objects or behaviors^46^. According to different application scenarios, the high-level (or low-level) layers are assumed to be consistent across tasks and can be shared for joint learning. For example, one study^47^ has proposed to fuse multi-modal neural imaging data by sharing high-level hidden layers. Imaging techniques, such as magnetic resonance imaging (MRI), functional MRI (fMRI) and positron emission tomography (PET) acquire data on different aspects of brain structure and function. Here, the low-level (pixel-level) hidden layers can carry modality-specific information, while the high-level layers aim to capture outcome (i.e., diagnosis)-related representations, that are thought to be consistent across modalities and thus shared between tasks. In this work, the authors successfully applied this strategy for predicting the diagnosis of Alzheimer’s disease. Besides images, deep MTL has been applied to other data modalities in biomedicine, e.g., natural language and DNA/RNA sequences. One study^48^ trained a convolutional neural model using text data from social media on the internet to identify associations with self-reported diagnoses of nine mental health conditions. Via the joint training, deep MTL achieved a superior prediction performance, especially on recall. Another recent study^49^ applied a multi-task multilayer perceptron on RNA-Seq data to integrate the prediction of six phenotypes related to Alzheimer’s disease. In all these applications, deep MTL models outperformed the models trained on a single modality.

In spite of the high prediction accuracy, there are two major challenges for applying deep MTL in biomedicine. First, a DNN model is difficult to interpret since hidden predictors are densely connected and ‘self-organized’. One possible solution is to apply a generative approach to DNN, i.e., deep belief networks (DBN)^50^. These networks allow the inference of the importance of each hidden parameter but are more difficult to train. Another challenge is the best selection of the network depth. There is no gold standard for the number of hidden layers, and too deep networks might require excessive computing time. Therefore, a common strategy is to pre-train the network layer-by-layer, followed by visualization of samples in the “transformed predictor space”. If the sample-outcome relationship is interpretable in transformed space (i.e., samples are linearly separable for classification), then the depth is sufficient. Otherwise, a new layer would be pre-trained and conditioned on the previous layers. Combining these techniques, a recent study^51^ applied deep MTL to integrate four sensorimotor prediction tasks. With systematic control of weight-sparsity levels across hidden layers, they obtained superior performance with few subjects.

**Step-by-step guidelines for MTL application**

To guide readers in applying MTL methods to their own data, we provide below a summary protocol describing the required steps from package installation to model interpretation. In the following sections, we explain this protocol based on actual code as well as examples. The code is based on the R package “*RMTL*”^52,53^. RMTL is a user-friendly software supporting the entire MTL analysis. The architecture of RMTL consists of an optimization procedure (bottom level), MTL algorithms (middle level) and auxiliary functions for the entire machine learning workflow (top level). As these elements were newly implemented, RMTL is not substantially dependent on other packages, which improves its adaptability to different computing environments.

Besides the simplified code shown in Box 1, a more detailed demonstration of the MTL workflow, RMTL algorithms and biological case studies can be found in the RMTL tutorial^54^ on CRAN (https://cran.r-project.org/). As an example, we will show readers the MTL analysis for selecting shared predictors, including model training, testing and parameter tuning. The code used is attached at the end of the section (**Box** **1**). **Supplementary figure 1** summarizes the suggested analysis protocol. After package installation and dataset preparation, users select one of the available algorithms and then test whether the selected algorithm captured the data well.


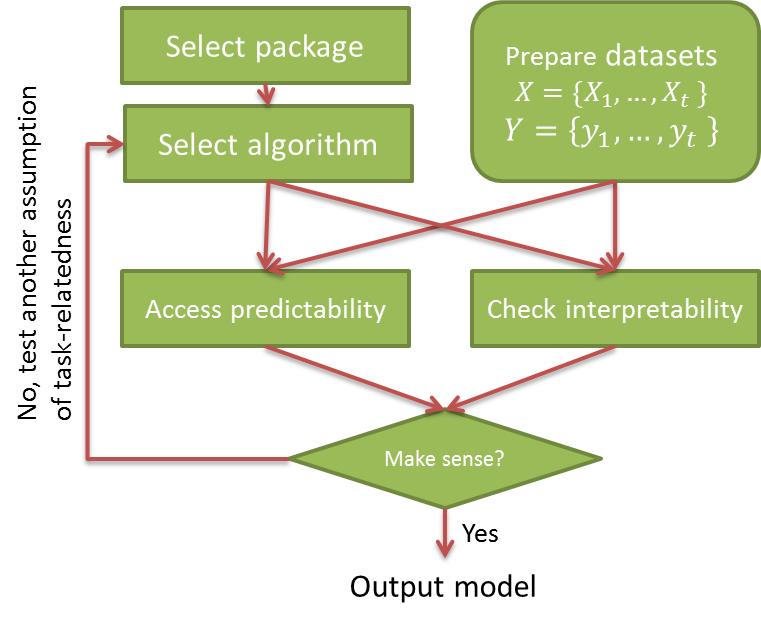


**Supplementary figure 1**. A step-by-step protocol for the application of MTL methods.

Prior to the analysis, as shown in Box 1, the simulation data was created with the default parameters^53^. Five regression tasks were created. Each task had 20 subjects and 50 features, and the top 4 features were the true signatures. The ground truth model can be found in **Supplementary Figure 2**.

Package installation

There are many publicly available examples of MTL code. However, most of these either contain only a single MTL method or do not allow comprehensive machine learning analysis. Therefore, we suggest that users utilize two MTL packages: RMTL^52,53^ and Malsar^55^. RMTL was written in the *R* environment and has been integrated into the CRAN platform (https://cran.r-project.org), facilitating straightforward installation. RMTL aims to provide a “simple-to-use” MTL framework, simplify the entire machine learning analysis, and is thus especially useful for investigators with limited programming and machine learning expertise. RMTL provides an efficient implementation of 10 MTL algorithms applicable for regression, classification, joint feature selection, task clustering, low-rank learning, sparse learning and network incorporation. Malsar was written in *Matlab*, contains more MTL algorithms than RMTL, but requires more advanced machine-learning expertise to conduct the analysis. Both packages implement solvers based on the accelerated proximal method and feature a state-of-art complexity of O(1/k^2).

Algorithm selection

To select a specific MTL method, users are encouraged to decide on a given task relationship prior to algorithm selection. According to the summary provided in **Supplementary** **Table 1** and domain knowledge regarding the investigated data, users may already have a prior assumption of the task relationship and a potentially useful regularization method. Such an assumption critically determines the potential success of the MTL analysis. With an incorrect specification of the task relationship, the prediction performance and model interpretability would most likely not be optimal. In RMTL, the utilization of different regularization strategies is convenient since all supported cross-task regularizations are aligned into one representation. To switch to another algorithm, users only need to change the name of the regularization. For example, in Box 1, users can change the assumed task relationship from “shared features” to “shared low dimensional space” by assigning a new string “Trace” to the variable “Regularization”, i.e., Regularization="Trace", and passing it to the training function.

Assessment of predictive performance

The measure of predictive performance differs depending on whether regression or classification is performed. RMTL supports the misclassification error for classification tasks and the mean squared error for regression tasks. To prevent overfitting and tune the appropriate parameters (i.e., $\lambda$ in **Supplementary Table 1**), RMTL integrates a cross-validation (CV) procedure. Cross-validation separates the training data into several folds with equal sample size, trains the MTL model on all but one fold and assesses predictive performance in the fold not used for training. This procedure is repeated until prediction is performed once for all folds. Based on this procedure, the optimal $\lambda$ is selected as that maximizing the predictive performance in the test folds. The $\lambda$ determines the strength of the penalty and reflects the structure of the assumed task relationship. For example, if cross-validation chooses a large $\lambda$ for MTL with joint predictor selection, a small set of shared predictors will be selected that can predict outcomes with maximum accuracy leading to a highly row-sparse coefficient matrix (**Supplementary figure 2b**). In **Supplementary figure 2a**, the CV error curve is shown, and the $\lambda$ with the lowest error is eventually selected for training. However, if there are multiple $\lambda$s corresponding to the same lowest error, then the largest $\lambda$ among those is selected due to the fewer number of selected features (the rule of Occam's Razor). This accuracy-driven approach for selecting $\lambda$ is effective for prediction tasks. However, in biomedical analysis, an important aspect is biological interpretability, in addition to the predictive capacity of a given solution. Therefore a more strict threshold of $\lambda$ might be more interesting because the features that are consistently associated with the outcomes given the sampling variability may contain valuable biological information.


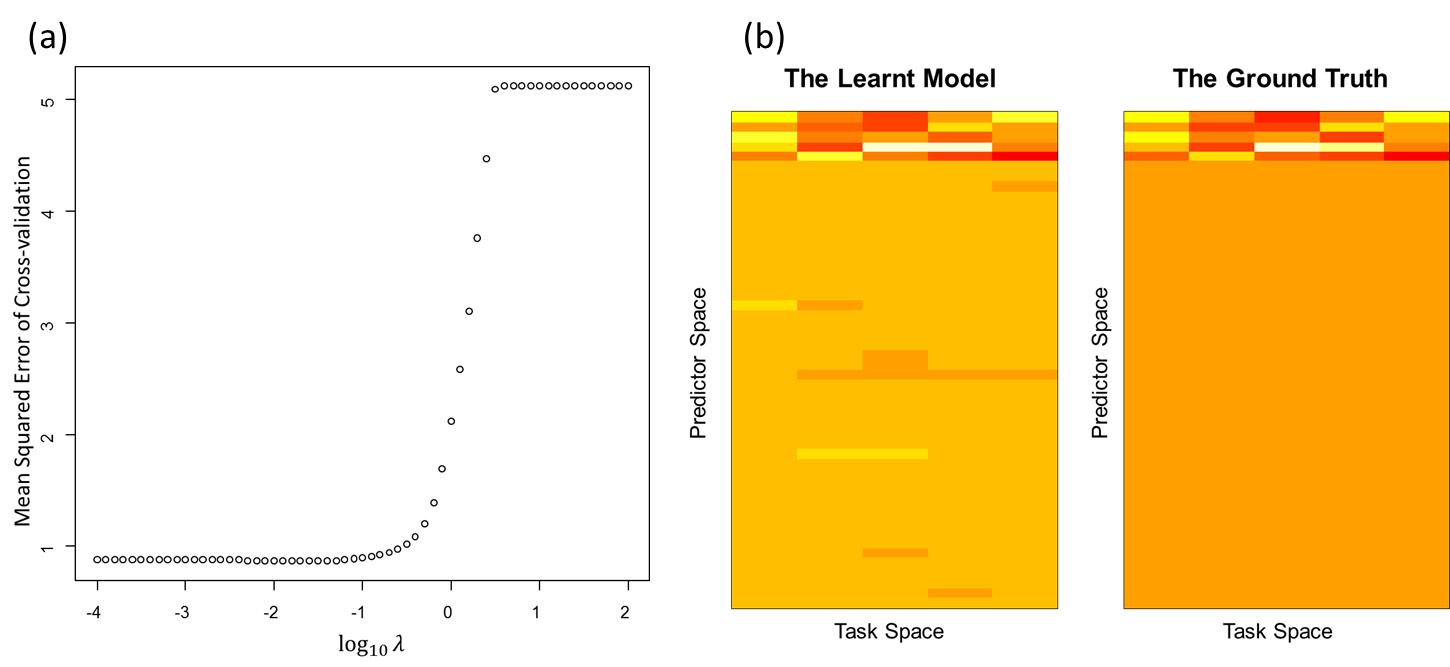


**Supplementary figure 2. Assessment of predictive performance and model interpretability for MTL with joint predictor selection.** (a) Prediction error during cross-validation. (b) Comparison of the row-sparse structure of the learnt model and the ground truth.

Assessment of model interpretability

Given the selected $\lambda$, users can train the MTL model using all training data for subsequent testing in independent test data. The interpretability of the model can be explored by investigating the coefficient matrix. Here we show this step for MTL with joint predictor selection. More examples can be found in the RMTL tutorial document^54^. **Supplementary figure 2b** displays the sparse structure of the learned coefficient matrix and the corresponding ground truth. The shared predictors were all well captured by the method. The true positive shared signals are well captured.

In addition to these steps, another consideration for using MTL in real analysis is to avoid circular analysis^56^. In MTL analysis, this refers to the same training subjects being repeatedly used in the workflow, leading to biased estimation. One common scenario is that the same training dataset was used for first selecting the $\lambda$ and then quantifying the prediction performance. The bias is introduced because the test data used during the performance assessment phase was already exposed to the model during the$\lambda$ selection phase. This bias will overestimate the prediction performance. This issue can be solved using resampling techniques. For example, the nested cross-validation can be used in MTL analysis, e.g., as shown in^57^, for unbias selection of $\lambda$ and quantification of prediction performance. This procedure repeatedly separates the dataset into discovery and holdout parts. And the holdout part of the data is used to validate the prediction performance and is also “unseen” by the $\lambda$ selection procedure.

############################################

#The MTL analysis to select shared predictors

#############################################

#Install package RMTL

install.packages("RMTL")

library("RMTL")

#Simulate datasets

set.seed(1)

data <- Create_simulated_data(Regularization="L21", type="Regression")

#Perform the cross-validation

set.seed(2)

cvfit<-cvMTL(data$X, data$Y, type="Regression", Regularization="L21",

Lam1_seq= 10^seq(2,-4,-0.1))

#Assess the CV prediction errors

plot(cvfit)

#Train on all training data

m=MTL(data$X, data$Y, type="Regression", Regularization="L21", Lam1=cvfit$Lam1.min, Lam1_seq=cvfit$Lam1_seq)

#Assess the prediction error on unseen data

paste0("test error: ", calcError(m, data$tX, data$tY))

#Compare the model to the ground truth

par(mfrow=c(1,2))

image(t(m$W), xlab="Task Space", ylab="Predictor Space")

title("The Learnt Model")

image(t(data$W), xlab="Task Space ", ylab="Predictor Space")

title("The Ground Truth")

**Box 1. Example code for MTL analysis based on the R package RMTL**

1. Tibshirani, R. Regression shrinkage and selection via the lasso. *Journal of the Royal Statistical Society. Series B (Methodological)*, 267-288 (1996).

2. Hoerl, A.E. & Kennard, R.W. Ridge Regression: Biased Estimation for Nonorthogonal Problems. *Technometrics* **12**, 55 (1970).

3. Zou, H. & Hastie, T. Regularization and variable selection via the elastic net. *Journal of the Royal Statistical Society: Series B (Statistical Methodology)* **67**, 301-320 (2005).

4. Meier, L., Van De Geer, S. & Bühlmann, P. The group lasso for logistic regression. *Journal of the Royal Statistical Society: Series B (Statistical Methodology)* **70**, 53-71 (2008).

5. Simon, N., Friedman, J., Hastie, T. & Tibshirani, R. A Sparse-Group Lasso. *Journal of Computational and Graphical Statistics* **22**, 231-245 (2013).

6. Tibshirani, R., Saunders, M., Rosset, S., Zhu, J. & Knight, K. Sparsity and smoothness via the fused lasso. *Journal of the Royal Statistical Society: Series B (Statistical Methodology)* **67**, 91-108 (2005).

7. Yang, T. *et al.* Absolute Fused Lasso and Its Application to Genome-Wide Association Studies. 1955-1964 (2016).

8. Yang, S. *et al.* Feature Grouping and Selection Over an Undirected Graph. *KDD*, 922-930 (2012).

9. Bondell, H.D. & Reich, B.J. Simultaneous regression shrinkage, variable selection, and supervised clustering of predictors with OSCAR. *Biometrics* **64**, 115-23 (2008).

10. Argyriou, A., Evgeniou, T. & Pontil, M. Multi-task feature learning. in *Advances in neural information processing systems* 41-48 (2007).

11. Ji, S. & Ye, J. An accelerated gradient method for trace norm minimization. in *Proceedings of the 26th annual international conference on machine learning* 457-464 (ACM, 2009).

12. Evgeniou, T. & Pontil, M. Regularized multi-task learning. in *Proceedings of the tenth ACM SIGKDD international conference on Knowledge discovery and data mining* 109-117 (ACM, 2004).

13. Zhou, J., Liu, J., Narayan, V.A. & Ye, J. Modeling Disease Progression via Fused Sparse Group Lasso. *KDD* **2012**, 1095-1103 (2012).

14. Zhou, J., Yuan, L., Liu, J. & Ye, J. A multi-task learning formulation for predicting disease progression. 814 (2011).

15. Zhang, Y. & Yeung, D.-Y. A convex formulation for learning task relationships in multi-task learning. *arXiv preprint arXiv:1203.3536* (2012).

16. Liu, S., Pan, S.J. & Ho, Q. Distributed Multi-Task Relationship Learning. 937-946 (2017).

17. Jacob, L., Vert, J.-p. & Bach, F.R. Clustered Multi-Task Learning: A Convex Formulation. in *Advances in Neural Information Processing Systems 21 (NIPS 2008)* (2008).

18. Zhou, J., Chen, J. & Ye, J. Clustered Multi-Task Learning Via Alternating Structure Optimization. in *Advances in Neural Information Processing Systems 24 (NIPS 2011)* 702-710 (2011).

19. Jalali, A., Sanghavi, S., Ruan, C. & Ravikumar, P.K. A dirty model for multi-task learning. in *Advances in neural information processing systems* 964-972 (2010).

20. Gong, P., Ye, J. & Zhang, C. Robust multi-task feature learning. in *Proceedings of the 18th ACM SIGKDD international conference on Knowledge discovery and data mining* 895-903 (ACM, 2012).

21. Romera-Paredes, B., Aung, H., Bianchi-Berthouze, N. & Pontil, M. Multilinear multitask learning. in *International Conference on Machine Learning* 1444-1452 (2013).

22. Lin, K., Xu, J., Baytas, I.M., Ji, S. & Zhou, J. Multi-Task Feature Interaction Learning. 1735-1744 (2016).

23. Parikh, N. & Boyd, S. Proximal algorithms. *Foundations and Trends® in Optimization* **1**, 127-239 (2014).

24. Beck, A. & Teboulle, M. A fast iterative shrinkage-thresholding algorithm for linear inverse problems. *SIAM journal on imaging sciences* **2**, 183-202 (2009).

25. Kohannim, O. *et al.* Discovery and Replication of Gene Influences on Brain Structure Using LASSO Regression. *Front Neurosci* **6**, 115 (2012).

26. Ashburner, M. *et al.* Gene ontology: tool for the unification of biology. The Gene Ontology Consortium. *Nat Genet* **25**, 25-9 (2000).

27. Biomarkers Definitions Working, G. Biomarkers and surrogate endpoints: preferred definitions and conceptual framework. *Clin Pharmacol Ther* **69**, 89-95 (2001).

28. Ma, S., Song, X. & Huang, J. Supervised group Lasso with applications to microarray data analysis. *BMC Bioinformatics* **8**, 60 (2007).

29. Zhou, H., Sehl, M.E., Sinsheimer, J.S. & Lange, K. Association screening of common and rare genetic variants by penalized regression. *Bioinformatics* **26**, 2375-82 (2010).

30. Lin, D. *et al.* Group sparse canonical correlation analysis for genomic data integration. *BMC Bioinformatics* **14**, 245 (2013).

31. Liu, J., Wang, K., Ma, S. & Huang, J. Accounting for linkage disequilibrium in genome-wide association studies: A penalized regression method. *Stat Interface* **6**, 99-115 (2013).

32. Cun, Y. & Frohlich, H. Biomarker gene signature discovery integrating network knowledge. *Biology (Basel)* **1**, 5-17 (2012).

33. Wu, Q. *et al.* Protein functional properties prediction in sparsely-label PPI networks through regularized non-negative matrix factorization. *BMC Syst Biol* **9 Suppl 1**, S9 (2015).

34. Li, C. & Li, H. Network-constrained regularization and variable selection for analysis of genomic data. *Bioinformatics* **24**, 1175-82 (2008).

35. Horst, R. & Thoai, N.V. DC Programming: Overview. *Journal of Optimization Theory and Applications* **103**, 1-43 (1999).

36. Avey, S. *et al.* Multiple network-constrained regressions expand insights into influenza vaccination responses. *Bioinformatics* **33**, i208-i216 (2017).

37. Krizhevsky, A., Sutskever, I. & Hinton, G.E. Imagenet classification with deep convolutional neural networks. in *Advances in neural information processing systems* 1097-1105 (2012).

38. Hinton, G. *et al.* Deep Neural Networks for Acoustic Modeling in Speech Recognition: The Shared Views of Four Research Groups. *IEEE Signal Processing Magazine* **29**, 82-97 (2012).

39. Collobert, R. & Weston, J. A unified architecture for natural language processing: deep neural networks with multitask learning. 160-167 (2008).

40. Jones, W., Alasoo, K., Fishman, D. & Parts, L. Computational biology: deep learning. *Emerging Topics in Life Sciences* **1**, 257-274 (2017).

41. Li, H., Habes, M. & Fan, Y. Deep ordinal ranking for multi-category diagnosis of alzheimer's disease using hippocampal MRI data. *arXiv preprint arXiv:1709.01599* (2017).

42. Deshpande, G., Wang, P., Rangaprakash, D. & Wilamowski, B. Fully Connected Cascade Artificial Neural Network Architecture for Attention Deficit Hyperactivity Disorder Classification From Functional Magnetic Resonance Imaging Data. *IEEE Trans Cybern* **45**, 2668-79 (2015).

43. Plis, S.M. *et al.* Deep learning for neuroimaging: a validation study. *Front Neurosci* **8**, 229 (2014).

44. Durstewitz, D., Koppe, G. & Meyer-Lindenberg, A. Deep neural networks in psychiatry. *Mol Psychiatry* (2019).

45. Ruder, S. An overview of multi-task learning in deep neural networks. *arXiv preprint arXiv:1706.05098* (2017).

46. Simonyan, K., Vedaldi, A. & Zisserman, A. Deep inside convolutional networks: Visualising image classification models and saliency maps. *arXiv preprint arXiv:1312.6034* (2013).

47. Shi, J., Zheng, X., Li, Y., Zhang, Q. & Ying, S. Multimodal Neuroimaging Feature Learning With Multimodal Stacked Deep Polynomial Networks for Diagnosis of Alzheimer's Disease. *IEEE J Biomed Health Inform* **22**, 173-183 (2018).

48. Cohan, A. *et al.* SMHD: A Large-Scale Resource for Exploring Online Language Usage for Multiple Mental Health Conditions. *arXiv preprint arXiv:1806.05258* (2018).

49. Beebe-Wang, N., Celik, S. & Lee, S.-I. MD-AD: Multi-task deep learning for Alzheimer's disease neuropathology. *bioRxiv*, 331942 (2018).

50. Hinton, G.E., Osindero, S. & Teh, Y.-W. A fast learning algorithm for deep belief nets. *Neural computation* **18**, 1527-1554 (2006).

51. Jang, H., Plis, S.M., Calhoun, V.D. & Lee, J.H. Task-specific feature extraction and classification of fMRI volumes using a deep neural network initialized with a deep belief network: Evaluation using sensorimotor tasks. *Neuroimage* **145**, 314-328 (2017).

52. Cao, H., Zhou, J. & Schwarz, E. RMTL: An R Library for Multi-Task Learning. *Bioinformatics* (2018).

53. Cao, H. & Schwarz, E. RMTL: Regularized Multi-Task Learning. (The Comprehensive R Archive Network, <https://cran.r-project.org/web/packages/RMTL/index.html>).

54. Cao, H. & Schwarz, E. An Tutorial for Regularized Multi-task Learning using the package RMTL. (The Comprehensive R Archive Network, <https://cran.r-project.org/web/packages/RMTL/vignettes/rmtl.html>).

55. Zhou, J., Chen, J. & Ye, J. MALSAR: Multi-tAsk Learning via StructurAl Regularization. (Arizona State University, 2012).

56. Kriegeskorte, N., Simmons, W.K., Bellgowan, P.S. & Baker, C.I. Circular analysis in systems neuroscience: the dangers of double dipping. *Nat Neurosci* **12**, 535-40 (2009).

57. Cao, H., Meyer-Lindenberg, A. & Schwarz, E. Comparative evaluation of machine learning strategies for analyzing big data in psychiatry. *International journal of molecular sciences* **19**, 3387 (2018).
